# A novel subgradient-based optimization algorithm for blockmodel functional module identification

**DOI:** 10.1186/1471-2105-14-S2-S23

**Published:** 2013-01-21

**Authors:** Yijie Wang, Xiaoning Qian

**Affiliations:** 1 Department of Computer Science and Engineering, University of South Florida, Tampa, FL 33620, USA

## Abstract

Functional module identification in biological networks may provide new insights into the complex interactions among biomolecules for a better understanding of cellular functional organization. Most of existing functional module identification methods are based on the optimization of network modularity and cluster networks into groups of nodes within which there are a higher-than-expectation number of edges. However, module identification simply based on this topological criterion may not discover certain kinds of biologically meaningful modules within which nodes are sparsely connected but have similar interaction patterns with the rest of the network. In order to unearth more biologically meaningful functional modules, we propose a novel efficient convex programming algorithm based on the subgradient method with heuristic path generation to solve the problem in a recently proposed framework of blockmodel module identification. We have implemented our algorithm for large-scale protein-protein interaction (PPI) networks, including *Saccharomyces cerevisia *and *Homo sapien *PPI networks collected from the Database of Interaction Proteins (DIP) and Human Protein Reference Database (HPRD). Our experimental results have shown that our algorithm achieves comparable network clustering performance in comparison to the more time-consuming simulated annealing (SA) optimization. Furthermore, preliminary results for identifying fine-grained functional modules in both biological networks and the comparison with the commonly adopted Markov Clustering (MCL) algorithm have demonstrated the potential of our algorithm to discover new types of modules, within which proteins are sparsely connected but with significantly enriched biological functionalities.

## Introduction

Biomolecules interact with each other in a complex modular manner to maintain normal cellular functionalities [1,2]. Identifying recurrent functional modules may help better understand the functional organization of cells [3]. Many existing network clustering algorithms for functional module identification focus on identifying "modules" within which nodes are densely connected [4-6]. However, identifying modules using these existing computational approaches may have artificially introduced biases from their module definitions and corresponding optimization methods [2]. Topologically defined modules may simply originate from the evolution process but do not necessarily correspond to functional units in cells [7]. In addition to densely self-connected modules, there are other topological structures in biological networks which capture important functional relationships among biomolecules. For example, transmembrane proteins, including receptors in many signal transduction pathways, have a special structure in which they rarely interact with each other but have a similar interaction patterns with the rest of the network [2]. To identify functional modules with richer topological structures, blockmodel network clustering recently has been proposed for functional module identification in biological networks [2,4,8,9].

Blockmodel module identification problem has been investigated for years [2,7,10] and has recently been used for blockmodeling functional modules within biological networks [2]. However, the resulting optimization problem is NP hard with highly nonlinear and non-convex properties with many local optima in its objective function. This makes it computationally prohibitive to obtain the optimal modules, especially for large-scale biological networks. Simulated Annealing (SA) algorithm has been used to solve the optimization problem [2]. However, a slow cooling down procedure is required to guarantee the solution quality. Furthermore, its computational time escalates quadratically with the increasing number of modules to identify. Therefore, more efficient algorithms are needed for discovering fine-grained functional modules in genome-scale biological networks.

In this paper, an efficient optimization method--subgradient with path generation(SGPG) is proposed to solve this difficult non-convex combinatorial optimization problem. In order to achieve results close to global optima, SGPG combines the convex programming method, which uses subgradient (SG) to efficiently obtain the local optima, and a heuristic path generation (PG) strategy, which makes use of the obtained local optima to search for better solutions. We have applied our SGPG as well as SA for functional module identification in two large-scale protein-protein interaction (PPI) networks: *Saccharomyces cerevisia *(*Sce*) PPI network from the Database of Interacting Proteins (DIP) [11] and *Homo sapien *(*Hsa*) PPI network collected from the Human Protein Reference Database (HPRD version 9) [12]. The results demonstrate that our new SGPG method achieves competitive performance numerically and biologically comparing to SA but with significantly reduced computation time. Furthermore, we have implemented SGPG and the Markov Clustering (MCL) algorithm [13] to find fine-grained modules of these two PPI networks. The results reveal that SGPG can identify additional biologically meaningful modules that MCL may miss, which may provide us a better understanding of the functional organization of these PPI networks.

### Blockmodel module identification

We first review the blockmodel module identification framework proposed in [2,7,10]. Any given network can be represented as a graph G={V,E}, where $V=\left\{v_{1},v_{2},\ldots ,v_{N}\right\}$ denotes the set of *N *network nodes in G , and E  is the set of edges. The topology of the network G  can be represented by an *N *× *N *adjacency matrix *A*, where each entry *A_ij _*represents the interaction between nodes *v_i _*and *v_j _*. The blockmodel framework introduces the image graph M={U,I} to abstract the function roles of the nodes in the original network and to outline the primary interactions among functional modules. In the image graph $M,\;U=\left\{u_{1},\ldots ,u_{q}\right\}$ represents the set of virtual module nodes in the module space and I preserves the interactions within U . The topology of M  also can be represented by a *q *× *q *adjacency matrix *B*, where the entry *B_rs _*denotes the interaction between modules *u_r _*and *u_s_*. For module identification, the mapping *τ *assigns *N *nodes in the original network G  to *q *different modules in the image graph M .

Assuming that we know the image graph adjacency matrix *B*, the optimization criterion of blockmodel framework is to minimize the mismatch between *A_ij _*and *B_rs _*by identifying an optimal mapping *τ *with resepct to the error function [2]:

(1) $$E\left(\tau ,\;B\right)=\frac{1}{M}\overset{N}{\underset{i\neq j}{\sum }}\left(A_{ij}-B_{\tau_{i}\tau_{j}}\right)\left(w_{ij}-p_{ij}\right),$$

in which *w_ij _*denotes the weight of the corresponding edge in G  (in this paper *w_ij _*= *A_ij_*); $M=\sum_{i\neq j}^{N}w_{ij}$ is used to restrict *E*(*τ*, *B*) between 0 and 1; and *p_ij _*denotes the penalty of mismatching for the corresponding absent edges, which can be determined by $p_{ij}=\frac{\Sigma_{k\neq i}w_{ik}\Sigma_{\iota \neq j}w_{lj}}{\Sigma_{k\neq l}w_{kl}}$[10].

In (1), we note that *E*(*τ*, *B*) = 0 when the image graph preserves all the edges of the original network $\left(B_{\tau_{i}\tau_{j}}=A_{ij}\right)$; Otherwise, *E*(*τ*, *B*) *>*0, meaning that the image graph does not preserve all the edges in the original network. When the image graph *B *has a mismatch for the absent edge between nodes *v_i _*and *v_j_*, it introduces error *p_ij_*; It contributes *w_ij _- p_ij _*to the error function in (1) when it has a mismatch for the existing edge between nodes *v_i _*and *v_j _*. Because *A_ij _*is constant, therefore, minimizing *E*(*τ, B*) is equivalent to maximizing $\frac{1}{M}\sum_{i\neq j}^{N}\left(w_{ij}-p_{ij}\right)B_{\tau_{i}\tau_{j}}$, which can be rewritten as ${\text{max}}_{\tau ,B}\frac{1}{2M}\sum_{i\neq j}^{N}\left(w_{ij}-p_{ij}\right)\left(2B_{\tau_{i}\tau_{j}}-1\right)$ by using the binary trick. With that, we can formulate the objective function as

$$Q\left(\tau ,\;B\right)=\frac{1}{2M}\underset{r,s}{\sum }\overset{N}{\underset{i\neq j}{\sum }}\left(w_{ij}-p_{ij}\right)\delta_{\tau_{i}r}\delta_{\tau_{j}s}\left(2B_{rs}-1\right),$$

where $\delta_{\tau_{i}r}$ is the indicator function that takes value 1 when *τ_i _*= *r *and 0 otherwise. In order to maximize *Q*(*τ*, *B*), *B_rs _*should be set to "1" when its corresponding term$\sum_{i\neq j}^{N}\left(w_{ij}-p_{ij}\right)\delta_{\tau_{i}r}\delta_{\tau_{j}\;s}$ is larger than 0, and 0 otherwise. Hence, the optimal solutions of *τ *and *B *are naturally decomposed. The optimal image graph *B *is a byproduct of the optimal mapping *τ *, which maximizes the following objective function:

(2) $$Q^{*}\left(\tau \right)=\frac{1}{2M}\overset{q}{\underset{r,s}{\sum }}\left|\overset{N}{\underset{i\neq j}{\sum }}\left(w_{ij}-p_{ij}\right)\delta_{\tau_{i}r}\delta_{\tau_{j}s}\right|\cdot$$

The optimization problem (2) is NP hard [2,14]. In [10], SA has been proposed to solve the optimization problem, which has the time complexity escalating with the increasing *q*. In our biological application, the search space for annealing parameters also increases with the increasing *q*. To find a large number *q *of functional modules in large-scale networks, SA is a very time-consuming algorithm.

### Subgradient with path generation (SGPG)

We propose to speed up the blockmodel module identification problem by convex programming combined with a heuristic path generation method. The basic idea is first to use the fast subgradient (SG) convex programming method to obtain the local optima, then use path generation (PG) to search for better solutions to reach global optima. We note that PG is originally proposed in this paper as a new useful heuristic to combine with subgradient algorithms to efficiently solve the hard combinatorial optimization problem. The combination of SG (time complexity *O*(*qN*^2^)) and PG (time complexity *O*(*q*^2^*N*^2^)) can dramatically reduce the computational time with competitive performance compared to SA method.

### Subgradient convex programming (SG)

#### Blockmodel module identification in matrix form

We now reformulate the module identification problem (2) into a matrix form by introducing an assignment matrix *S *corresponding to the module mapping *τ*. To identify *q *non-overlapping functional modules in G , the assignment matrix *S *is defined as an *N *× *q *matrix with each entry *S_ir _*= 1 when *v_i _*is assigned to the module *u_r _*or equivalently, *τ_i _*= *r*; and *S_ir _*= 0 otherwise. In other words, $S_{ir}=\delta_{\tau_{i}r}$. Each column in *S *corresponds to an image graph module node in which all the assigned network nodes take the value "1". We further use *W *to denote the weight matrix with each entry as the corresponding edge weight *w_ij_*, and *P *as the penalty matrix with each entry as the corresponding penalty *p_ij_*. The objective function in (2) can be rewritten in the following equivalent matrix form:

(3) $$Q^{*}\left(\tau \right)=f\left(S\right)=\;{∥S^{T}\left(W-P\right)S∥}_{L_{1}},$$

in which the sum of each row in *S *has to be the unity and the columns of *S *are orthogonal to each other. In addition, if we assume that each node has to be assigned to one module, the assignment matrix *S *has to satisfy the normalization condition *S*1*_q _*= 1*_N_*, in which 1*_q _*and 1*_N _*denote the *q*-dimensional and *N*-dimensional vectors of all ones. Hence, the optimal solution for the assignment matrix *S *lies in the space $\phi =\left\{S\in {\left\{0,1\right\}}^{N\times q},\;S1_{q}=1_{N}\right\}$, we have the convex programming formulation:

(4) $$\begin{array}{c} \min_{S}:F\left(S\right):=-{∥S^{T}\left(W-P\right)S∥}_{L_{1}}\\ s.t.\;\;\;S\in \phi . \end{array}$$

Note that we have converted our maximization problem into a minimization problem for the convenience of introducing subgradient methods in convex programming [15]. We denote *Q *= *S^T ^*(*W *- *P*) *S *with its entries $Q_{rs}=S_{r}^{T}\left(W-P\right)S_{s}$, where *S_r _*is the *r*th column of *S*. Again, with the optimal assignment matrix *S*, we can derive the topology of the image graph *B*: *B_rs _*= 1 if *Q_rs _>*0, and 0 otherwise.

#### Subgradient

The optimization problem (4) is a non-smooth combinatorial optimization problem as the objective function involves the *L*_1 _norm of the matrix *Q*. To solve this hard optimization problem, we first relax the binary constraints $S\in {\left\{0,1\right\}}^{N\times q}$ in (4) by the continuous relaxation $S\in {\left[0,1\right]}^{N\times q}$ and use *γ *to represent the relaxed constraint set, which is a convex hull after relaxation. To address the nonlinearity of the matrix *L*_1 _norm objective function $F\left(S\right)=-{∥Q∥}_{L_{1}}$ with the relaxed linear constraints, we propose to use Frank-Wolfe algorithm [15] to iteratively solve the following optimization problem with a linear objective function from the approximation by the first-order Taylor expansion:

(5) $$\begin{array}{c} {\text{min}}_{S}:F\left(S^{t}\right)+<\nabla F\left(S^{t}\right),\left(S-S^{t}\right)>\\ s.t.\;\;\;S\in \gamma , \end{array}$$

where *S^t ^*is the current solution, <, > is the inner product operator, and the new objective function is from the first-order Taylor expansion. The problem (5) at each iteration is a linear programming problem to search for the local extreme point along the gradient ∇*F*(*S^t^*) as in steepest descent. However, as previously stated, *F*(*S^t^*) takes the matrix *L*_1 _norm, which is non-smooth, and therefore non-differentiable. To address this last complexity, we apply subgradient methods [15] to replace ∇*F*(*S^t^*) by a subgradient *∂F*(*S^t^*) instead [16]:

**Definition (Subgradient): **A matrix $\partial F\in R^{N\times q}$ is a subgradient of a function $F:R^{N\times q}\to R$ at the matrix $X\in R^{N\times q}$ if $F\left(Z\right)\geq F\left(X\right)+<\partial F,\left(Z-X\right)>,\forall Z\in R^{N\times q}$.

In our case, the subgradient of the matrix *L*_1 _norm can be presented by its dual norm--matrix *L_∞ _*norm, which is used to derive the subgradient *∂F*(*S^t^*). Similar to the derivation for the subgradient of the *L*_1 _norm of vectors in *L*_1 _regularization in [16], we show that the subgradient of the *L*_1 _norm of any matrix $X_{N\times q}$ is

(6) $$\partial ||X|{|}_{L_{1}}=\left\{\begin{array}{cc} \left\{\left(Y\;\in \;R^{N\times q};||Y|{|}_{L_{\infty }}\leq 1\right)\right\} & \text{if}\;X=0;\\ \left\{Y\;\in \;R^{N\times q};||Y|{|}_{L_{\infty }}\leq 1\;\;and\;<\;Y,\;X\;>\;=\;||X|{|}_{L_{1}}\right\}, & \text{otherwise;} \end{array}\right)$$

where **0 **is a *N *× *q *matrix of all zeros. For our module identification problem, we have the following proposition derived from (6):

**Proposition: **The subgradient of the objective function of our relaxed optimization problem *F *(*S*) at the assignment *S^t ^*can be defined as: $\partial F\left(S^{t}\right)=2\left(P-W\right)S^{t}\bar{Q}$. In our implementation, we choose

(7) $${\bar{Q}}_{rs}=\left\{\begin{array}{cc} \alpha & Q_{rs}=0;\\ 1 & Q_{rs}>0;\\ -1 & Q_{rs}<0; \end{array}\right)$$

where *α *is a number between [-1, 1].

**Proof: **From (6), there always exists a $\bar{Q}$ satisfying ${∥\bar{Q}∥}_{L_{\infty }}\leq 1$ and ${∥Q∥}_{L_{1}}=<\bar{Q},Q>$. As $\partial {∥Q∥}_{L1}=\partial <\bar{Q},Q>$ and the subgradient of differentiable functions is equal to its gradient [16], we have $\partial F\left(S^{t}\right)=-\partial \;\left[||Q|{|}_{L1}\right]=-\partial <\bar{Q},Q>=-\partial \text{tr}\left({\bar{Q}}^{T}S^{t^{T}}\left(W-P\right)S^{t}\right)=2\left(P-W\right)S^{t}\bar{Q}$ when *S^t ^*is close to the local minima. QED.    □

#### Convex programming algorithm

Using Frank-Wolfe algorithm with the derived subgradient, we now have a conditional subgradient method [16] to iteratively solve the relaxed optimization problem as shown in the pseudo-code given in the following:.

Algorithm: Conditional Subgradient

**Input: **initial value *S^t^*, *t *= 0.

Do:

      **(i) **Compute the subgradient *∂F *(*S^t^*).

      **(ii) **Solve the minimization problem:

            S* = arg min*_S _*: <*∂F *(*S^t^*), *S *>*s.t. S *∈ γ

      **(iii) **Linear search for the step in the direction S* - *S^t ^*found in (ii), update *S^t^*, *t *= *t *+ 1.

**Until: **|Δ*F*| + ||Δ*S^t^*|| <*ζ*

**Output: ***S^t^*.

In this algorithm, step (ii) at each iteration can be solved using a generic linear programming solver in *O*((*qN *)^3.5^). However, due to the special structure of the optimization problem, we instead solve it as a semi-linear assignment problem. As the assignment matrix [*∂F*(*S^t^*)]*_N × q _*is not a square matrix, the optimization in step (ii) can be efficiently solved by assigning node *i *to module *r*, which is the index of the largest entry in row *i *of subgrident *∂F*(*S^t^*), with the time complexity *O*(*qN*).

To derive the solution to the original problem (4) from the results of the relaxed problem by the conditional subgradient algorithm, we recover from the relaxed solution to a closest feasible solution by a simple rounding up strategy. Finally, we note that the presented conditional subgradient algorithm converges to a local stationary point of the combinatorial optimization problem (4) due to the non-convex nature of the objective function (3) with the worst case complexity *O*(*qN*^2^) [15]. Hence, good initialization is critical to get high quality results. In our current implementation, we initialize *S^t ^*by a modified Expectation-Maximization (EM) algorithm presented in [8].

### Path generation (PG)

In order to make use of the local optima found by the above fast subgradient method, we propose a novel path generation method for our combinatorial optimization problem. The path generation method aims to conserve the overlap between two local optima, and get improvement based on the overlap which contributes significantly to the objective function value. Our new path generation is inspired by the path relinking method which connects two combinatorial local optima and try to find better results along the linking path [14]. However, our method does not relink two local optimal results but creates new paths by extracting potentially useful overlap between them.

The essential idea of the path generation method is to construct new results by preserving the overlap between modules from two local optima that contributes significantly to the objective function. Given two solutions *x_A _*and *x_B _*from SG as the new path generators, PG generates new results and explores the search space while maintaining the current productive overlap between *x_A _*and *x_B_*. Let *N_r_*(*x_A_*) denote the module *u_r _*of *x_A _*and *N_s_*(*x_B_*) the module *u_s _*of *x_B_*. The contribution *S*(*r_A_*, *s_B_*) from the overlap *Over*(*r_A_*, *s_B_*) = {*d|d *= *N_r_*(*x_A_*) *∩ N_s_*(*x_B_*)} is defined as:

(8) $$S\left(r_{A},\;s_{B}\right)=\;{∥s_{AB}^{T}\left(W-P\right)S_{A}∥}_{L1}+{∥s_{AB}^{T}\left(W-P\right)S_{B}∥}_{L1}.$$

Here, *s_AB _*is a binary vector, of which each entry is equal to 1 when the corresponding node is in both *N_r_*(*x_A_*) and *N_s_*(*x_B_*), and 0 otherwise. The value of *S*(*r_A_, s_B _*) is the shared contribution to the objective function Q* in (2) between *N_r_*(*x_A_*) and *N_s_*(*x_B_*) for two feasible solutions. *S_A _*and *S_B _*are assignment matrix of the two solutions. The most promising overlap between modules *r *and *s *are determined by

(9) $$\left(r_{A},\;s_{B}\right)=argmax\left\{S\left(r,\;s\right):r,\;s\in \left\{1,\;\ldots ,\;q\right\}\right\}.$$

The path generation based on (9) proceeds in the following manner: First, the most promising overlap *Over*(*r_A_, s_B_*) between modules *r_A _*and *s_B _*of the initiating solution *x_A _*and the guiding solution *x_B _*is identified by (9), then *r_A _*is locally adjusted to become *Over*(*r_A_*, *s_B_*) by removing nodes. After the adjustment, a new solution *x*_1 _is generated and *C_A _*= {*r_A_*} and *C_B _*= {*s_B_*}, where *C_A _*and *C_B _*denote the sets of used modules in both solutions, respectively. Local search is then applied to find the improved $x_{1}^{*}$. Then we preserve $x_{1}^{*}$ and let $x_{A}=x_{1}^{*}$. The above procedure is repeated until no overlap exists or it reaches other relaxed termination conditions, for example, we can set *N_stop _*= 5 meaning that there are no larger than five nodes in the overlap of the modules from two solutions. Finally, we obtain the best solution along the generated paths. The whole procedure is illustrated in the following pseudo code:

Algorithm: Path Generation Method

**Input: ***x_A_*, *x_B_*, *x*, *x_best_*, *N_stop_*, *C_A _*= Ø, *C_B _*= Ø, *Over *= +*∞*, *Q_best _*= *−∞*

**While(***Over > N_stop_***)**

**(1)**      (*r_A_*, *s_B_*) = *argmax*{*S*(*r*, *s*): *r*, *s *∈ {1, ..., *q*} } and find *Over*(*r_A_*, *s_B _*);

**(2)**      modify nodes from *r_A _*in *x *to make *N_r _*(*x*) = *Over*(*r_A_*, *s_B_*) and *C_A _*= {*r_A_*}, *C_B _*= {*s_B_*};

**(3)**      $\left(Q_{x}^{*},x^{*}\right)$ = LocalSearch(*x*);

**(4)**      If $\left(Q_{x}^{*}>Q_{best}\right)$

**(5)**            *Q_best _*= $Q_{x}^{*}$ and *x_best _*= x*;

(6)      EndIf

**(7)**      *x_A _*= x* and find the next *Over *set using (9);

EndWhile

**Output: ***x_best _*and *Q_best_*.

**Figure 1:**
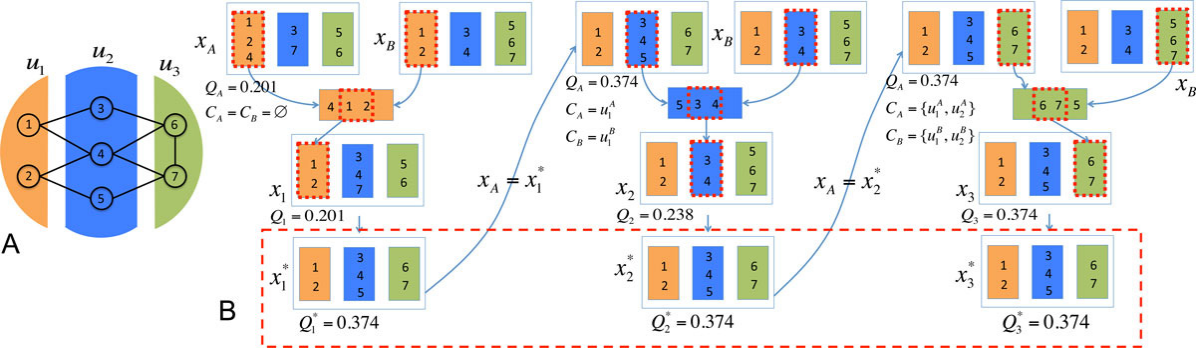
**An example of path generation: A. Network structure; B. Path generation procedure**.

To illustrate how PG works, an example of the path generation procedure is shown in Figure 1. The module organization of the given network is shown in Figure 1A. Assume *x_A _*= {{1, 2, 4}, {5, 6}, {3, 7}} with $Q_{x_{A}}^{*}=0.201$ and *x_B _*= {{1, 2}, {3, 4}, {5, 6, 7}} with $Q_{x_{B}}^{*}=0.238$. Starting with *C_A _*= *C_B _*= Ø, a path is generated. At the first step, the most productive overlap between module $r_{A}=u_{1}^{A}$ in *x_A _*and $s_{B}=u_{1}^{B}$ in *x_B _*is identified, and a new solution *x*_1 _is obtained by modifying $r_{A}=u_{1}^{A}$ to be the same as *Over*$\left(u_{1}^{A},\;u_{2}^{B}\right)$ with *Q*_1 _= 0.201. Update $C_{A}=\left\{u_{1}^{A}\right\}$ and $C_{B}=\left\{u_{1}^{B}\right\}$. Local search further improves the solution to obtain $x_{1}^{*}$ with $Q_{1}^{*}=0.374$ and then set $x_{A}=x_{1}^{*}$. Next, module $r_{A}=u_{2}^{A}$ and $s_{B}=u_{2}^{B}$ have the overlap with the largest contribution to the objective function. By similarly modifying $N_{2}\left(x_{A}\right)=Over\left(u_{2}^{A},\;u_{2}^{B}\right)$, we then generate a path $x_{2}^{*}$ with $Q_{2}^{*}=0.374$ and update $C_{A}=\left\{u_{1}^{A},\;u_{2}^{A}\right\}$ and $C_{B}=\left\{u_{1}^{B},\;u_{2}^{B}\right\}$. In the end, we make $N_{3}\left(x_{A}\right)=Over\left(u_{3}^{A},\;u_{3}^{B}\right)$ and get the final path $x_{3}^{*}$ with $Q_{3}^{*}=0.374$ and update $C_{A}=\left\{u_{1}^{A},\;u_{2}^{A},\;u_{3}^{A}\right\}$ and $C_{B}=\left\{u_{1}^{B},\;u_{2}^{B},\;u_{3}^{B}\right\}$. The PG algorithm is executed as in Figure 1 with the time complexity *O*(*q*^2^*N*^2^).

### Experimental results

We have implemented our SGPG method to identify functional modules in two biological networks: *Saccharomyces cerevisia *PPI network from the Database of Interacting Proteins (DIP) [11] and *Homo sapien *PPI network from the Human Protein Reference Database(HPRD) [12]. We first show the efficiency of SGPG comparing to the previous algorithms based on SA for functional module identification in two networks with *q *= 10, 50 and 100. We further evaluate the potential of SGPG to identify biologically meaningful modules by contrasting the differences of the identified fine-grained modules (*q *= 500 for *Homo sapien *PPI network and *q *= 300 for *Saccharomyces cerevisia *PPI network) detected by MCL algorithm [13]. We show that SGPG can unearth certain kinds of biologically meaningful modules that may not be detected by MCL.

### Performance comparison between SA and SGPG

We first compare SA and SGPG for module identification in two PPI networks with relatively small *q *= 10, 50, and 100 as SA requires very slow cooling down procedures to guarantee the solution quality when *q >*100. The *Homo sapien *PPI network has a largest component of 9,270 nodes and 36,917 edges. The upper bound of the objective function value in (2) $Q_{max}^{*}=0.98$ when we consider the original network itself as the image graph with *q *=9,270. We also have implemented our algorithm to the *Saccharomyces cerevisia *PPI network, which has a largest component of 4990 nodes and 21,911 edges with the upper bound $Q_{max}^{*}=0.\text{97}$ when *q *=4,990.

The parameter settings for SA and SGPG are listed in Table 1. The starting temperature and cooling down procedure are two critical parameters that determine the performance of SA. In our implementation, the starting temperature is set high enough and the cooling down procedure is set slow enough to avoid freezing in metastable states. For SGPG, we set the number of local optima *N_set _*to 10 and the terminal condition *N_stop _*= 5 for the minimum requirement of the overlap set *Over *in path generation.

**Table 1:** Parameter settings in SA and SGPG

**Para**.	*C_β_*	*T_start_*	*T_end_*	*T_sweep_*	*T_switch_*	*N_set_*	***N_stop_***.
SA	0.99	40	0.001	100	20	-	-
SGPG	-	-	-	-	-	10	5

Table 2 shows the comparison of the fitting score Q* computed by (2) and the running time between SA and SGPG. Because there is no ground truth of the exact functional modules in both networks, we use the fitting score Q* as the criterion of the optimization performance. All the experiments are programmed in C++ and run on a MacPro Station with a 2.4 GHz CPU and 6 Gb RAM. From Table 2 we find that the quality of the solutions computed by SGPG is very competitive to the solutions of SA with the largest gap 3.9% in Q* when *q *= 100 for *Homo sapien *PPI network. Meanwhile, SGPG is significantly faster than SA. Table 2 also reveals that the computation time of SA grows quadratically with increasing *q*, however, the computation time of SGPG grows sub-quadratically with *q *since the time complexity of SG and PG are *O*(*qN*^2^) and *O*(*q*^2^*N*^2^) respectively. This makes a big difference when we need to identify a large number of modules. For example, SA takes more than two months for detecting *q *= 300 modules for the *Homo sapien *PPI network, while SGPG only requires around two days to obtain the results.

**Table 2:** Comparison of SA and SGPG on *Homo sapien *and *Saccharomyces cerevisia *PPI networks

PPI	Method	Q*(*q*=10)	Time(h)	Q*(*q*=50)	Time(h)	Q*(*q*=100)	Time(h)
*Homo sapien*	SA	0.5393	1.73	0.6530	45.07	0.7180	180.26
	SGPR	0.5346	0.5	0.6452	1.95	0.6898	6.35

*Saccharomyces cerevisia *	SA	0.5692	1.35	0.6834	25.02	0.7544	102.65
	SGPR	0.5690	0.3	0.6752	1.15	0.7292	3.34

**Figure 2:**
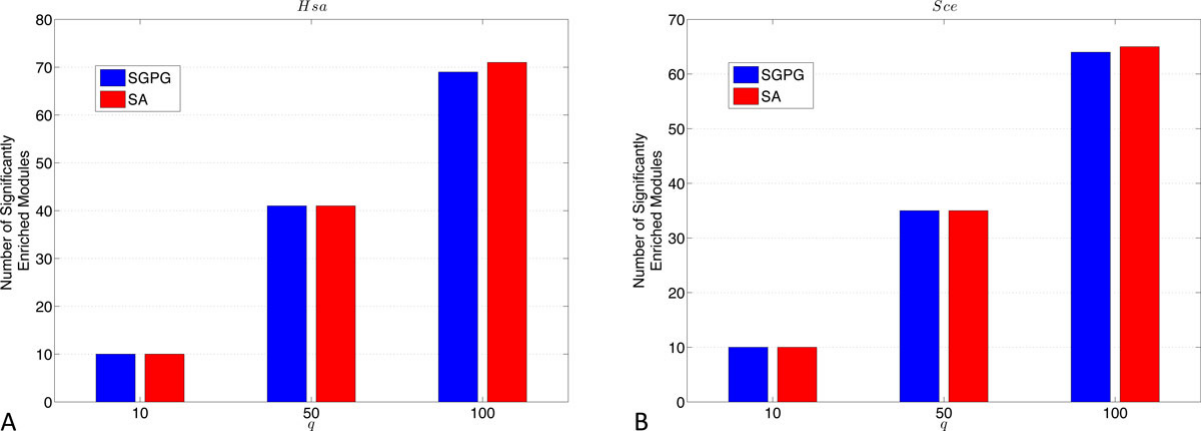
**Comparison between SA and SGPG for the number of identified modules of *Homo sapien *PPI network (A) and *Saccharomyces cerevisia *PPI network (B) that have significantly enriched GO terms below 1% after Bonferroni-correction in either biological process, molecular function, or cellular compartment**.

To further demonstrate whether these two different blockmodel methods have the potential of discovering biologically meaningful modules, we perform Gene Onotolgy (GO) enrichment analysis for the modules identified by both methods using GoTermFinder [17]. Figure 2 displays the comparison of the number of significantly enriched modules with different *q *detected by both SA and SGPG. From both figures, we find that SGPG achieves competitive performances on identifying GO enriched modules comparing to SA.

### Comparison between SGPG and MCL

In order to verify the biological significance of the modules identified by blockmodel identification, we have implemented both SGPG and MCL to detect fine-grained modules for both *Saccharomyces cerevisia *and *Homo sapien *PPI networks. Because SA will take months to obtain results with *q >*200, we only have applied SGPG in this section. By analyzing the identified modules detected by two methods, we have found that SGPG can discover a comparable number of GO enriched modules as MCL detects. More importantly, SGPG discovers additional biologically meaningful modules in which proteins are sparsely connected but have the same interaction patterns to the rest of the network.

#### Saccharomyces cerevisia PPI network

We have identified fine-grained modules for the *Saccharomyces cerevisia *PPI network using SGPG and MCL. We set *q *= 300 for SGPG and the inflation parameter *I *= 1.5 for MCL, which identified 370 modules in total. Within these identified modules, 296 modules by SGPG and 307 modules by MCL have more than two nodes. From these, we have found 150 and 153 modules respectively with significantly enriched GO terms below 1% after Bonferroni-correction by GoTermFinder. SGPG performs competitively to MCL. But more importantly, we find that SGPG can detect sparsely connected modules with certain interaction patterns that MCL fails to detect.

**Figure 3:**
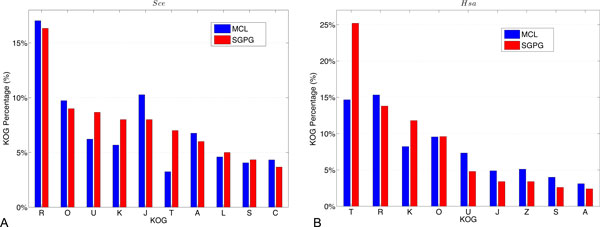
**Percentage of different categories of modules detected by SGPG and MCL (annotated by KOG)**. A. KOG percentage of *Saccharomyces cerevisia *PPI network. B. KOG percentage of *Homo sapien *PPI network.

In order to scrutinize the differences between the modules discovered by SGPG and MCL. We have annotated all modules by KOG categories [18]. For each module, the most KOG category assigned to the proteins in the module is annotated to that module. Figure 3A displays the percentage of the KOG annotated modules. Obviously, the percentages of modules annotated to KOG U, K, J and T are different (difference is larger than 2.5%) for the results from two methods. Specifically, SGPG detects more modules with KOG U, K and T annotations. To further examine the functionalities of different KOG categories, we discover that proteins annotated to KOG U play roles in intracellular trafficking, secretion, and vesicular transport; proteins annotated to KOG K have functionalities in transcription; and proteins in KOG T behave signal transduction functionalities. In [2], the results have already illustrated that proteins annotated to KOG T and K have the sparsely connected modules structures. Blockmodel based SGPG has successfully discovered more such modules than MCL. For proteins in KOG J (functions in translation, ribosomal structure and biogenesis), they are supposed to have densely connected modular structures which MCL tends to detect.

To verify that SGPG does detect sparsely connected modules, we investigate the network topology of the proteins of all the modules annotated to KOG U, K, J and T. We count the number of sparsely connected modules, which have the interaction density of module less than 3%, annotated to KOG U, K, J and T. The specific comparison is in Table 3. Table 3 illustrates the difference of network topology by the average module density and the average clustering coefficient among proteins detected by both SGPG and MCL. The average clustering coefficients of the induced network, which is extracted from the original *Saccharomyces cerevisia *PPI network based on the proteins of modules annotated to certain KOG categories, are calculated by the definition in [19]. Larger average clustering coefficients indicate that proteins have densely connected modular structures [19]. From the table, we find there is a trend that the average module density and the average clustering coefficient of the modules identified by MCL are larger than those detected by SGPG, which means MCL detects more densely connected modules while SGPG can identify sparsely connected modules.

**Table 3:** Topological analysis of different KOG categories in *Saccharomyces cerevisia *PPI network

KOG ID	Method	proteins	sparse modules/modules	Avg. density	**Avg. clustering coef**.
U	SGPG	353	15/26	2.98%	0.0814
	MCL	256	0/21	27.38%	0.2402

K	SGPG	359	6/24	6.68%	0.1352
	MCL	361	0/19	26.35%0	0.1834

J	SGPG	579	9/24	9.16%	0.0678
	MCL	358	0/25	37.90%	0.1429

T	SGPG	169	13/21	3.47%	0.0755
	MCL	94	0/12	31.31%	0.0912

**Figure 4:**
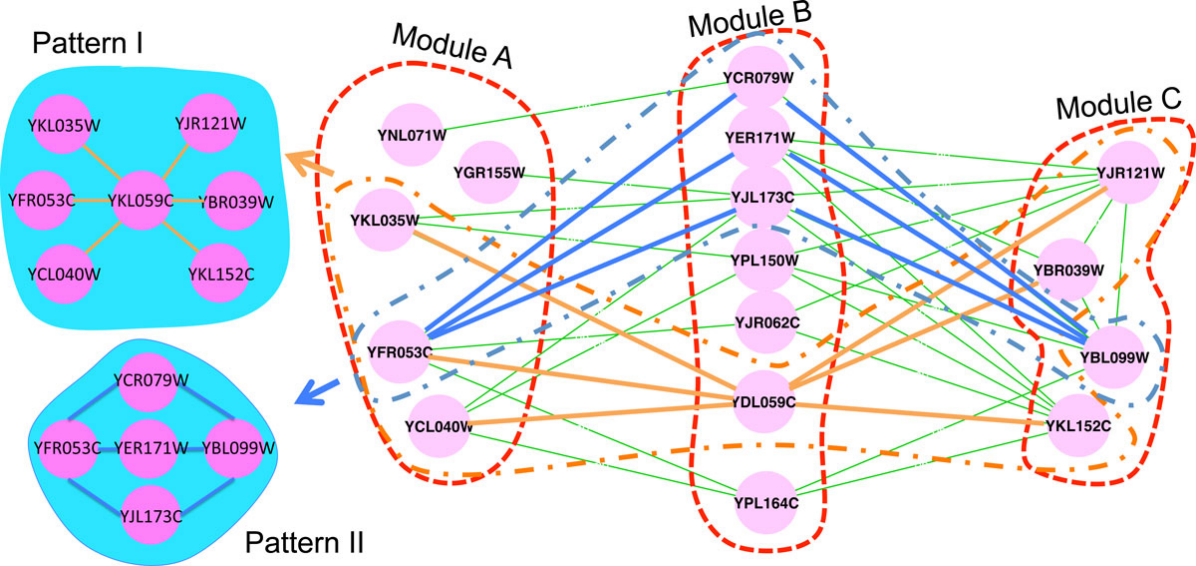
**A subnetwork with sparsely connected modules detected by SGPG**. Module A is enriched in hexokinase activity with p-value 1.71e-5. Module B is enriched in response to endogenous stimulus with p-value 4.77e-5. Module C is enriched in nucleoside phosphate metabolism with p-value 3.43e-6. Patterns I and II are two specific interaction patterns in the subnetwork.

Figure 4 illustrates an induced subnetwork of sparsely connected modules discovered by SGPG from the *Saccharomyces cerevisia *PPI network. Only the interactions among the proteins in Figure 4 are displayed. As shown in Figure 4, modules A and B are both sparsely connected modules, in which there is no interaction among proteins. Module C is a topologically cohesive module. Modules A, B and C are all significantly enriched in GO terms related to KOG G (carbohydrate transport and metabolism), T (signal transduction mechanisms) and C (energy production and conversion) respectively. From the structure illustrated in Figure 4, we find that proteins in module B play roles in passing signal between proteins of hexokinase activity and nucleoside phosphate metabolism. Furthermore, we notice that the marked patterns I and II are two types of interaction patterns across these modules, which tend to be clustered into the same module when using MCL. Figure 4 clearly displays the advantage of SGPG, which is to identify modules by their interaction patterns and functional roles rather than their interaction density.

Additionally, Table 4 illustrates three sparsely connected module examples including module B in Figure 4, which are all detected by SGPG but missed by MCL. These three modules are annotated to KOG U and T respectively, which cannot be detected by MCL no matter what inflation parameter we choose.

**Table 4:** Sparse modules in U and T KOG categories for *Saccharomyces cerevisia *PPI network

KOG ID	Sparse module example	Enriched genes	Enriched GO Term	GO Level	*p*-value
U	YDR179C, YNL287W, YDL216C YCR099C, YIL004C,YAL026C YLR268W, YLR093C, YPR163C YPR148C, YOL064C, YOL117W YGL084C, YLR031W, YIL076W YPL179W, YKL191W, YPL010W	YOL117W, YDR179C, YDL216C	protein deneddylation	[+8, 0]	2.01e-5

T	YJL092W, YDR490C, YOR231W YJL005W, YPL074W, YPL083C YNL323W,YOL100W	YDR490C, YOL100W, YNL323W, YJL005W, YOR231W	signal transduction	[+3, -1]	6.09e-5

T	YDR076W, YDL059C, YJL173C YPL164C, YER171W, YPL026C YCR079W, YPL150W, YHR169W YJR062C	YDL059C, YPL026C, YER171W, YPL164C, YJL173C, YDR076W	response to endogenous stimulus	[+2, -1]	4.77e-5

#### Homo sapien PPI network

For the *Homo sapien *PPI network, we set SGPG to identify *q *= 500 modules with the same settings in Table 1. For MCL, we set its inflation parameter *I *= 1.5 and have found 450 modules. We have performed GO enrichment analysis for these identified modules with more than two nodes (478 from SGPG and 380 from MCL). Based on GoTermFinder, 269 modules from SGPG and 265 modules from MCL are significantly enriched with p-values below 1% after Bonferroni-correction. SGPG has discovered a competitive number of GO enriched modules compared to MCL. We also note that the modules identified by SGPG are relatively smaller than those from MCL and these modules have more specific enriched functionalities and may provide more detailed information for future catalog of functional modules. More importantly, SGPG detects several modules with interesting functionalities that MCL has missed.

Following the same analysis method used in the previous section, we first annotate all the identified modules with KOG categories to scrutinize the differences between modules detected by SGPG and MCL. Figure 3B shows the percentages of the modules annotated to different KOG categories by both methods. Obviously, SGPG detects more modules annotated in KOG T and K categories, within which functional modules tend to have sparsely connected structures. However, MCL discovers more modules annotated in KOG U, within which functional modules tend to have a densely connected structure in the *Homo sapien *PPI network.

Table 5 further consolidates that the modules detected by SGPG can detect sparsely connected patterns that MCL may miss. The average density and average clustering coefficient both indicate that modules discovered by MCL have cohesive modular structure, while modules discovered by SGPG are sparsely connected.

**Table 5:** Topological analysis of different KOG categories in *Homo sapien *PPI network

KOG ID	Method	proteins	sparse modules/modules	Avg. density	**Avg. clustering coef**.
T	SGPG	1970	59/126	4.91%	0.0822
	MCL	2481	0/66	26.32%	0.1696

K	SGPG	878	27/59	3.15%	0.0779
	MCL	916	0/37	30.41%0	0.1928

U	SGPG	592	3/24	4.95%	0.0448
	MCL	517	0/33	31.42%	0.1359

**Figure 5:**
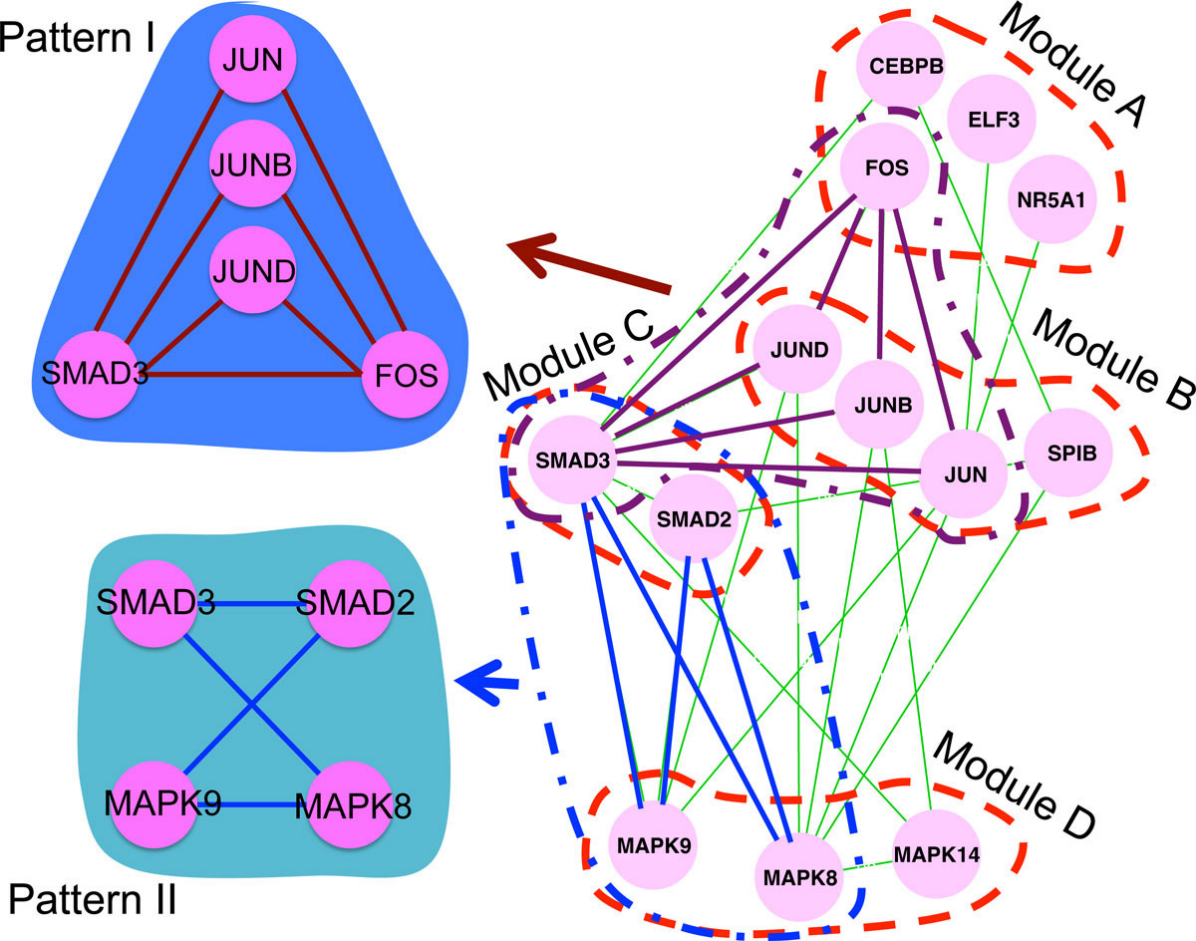
**A subnetwork with sparsely connected modules detected by SGPG**. Module A is enriched in sequence-specific DNA binding with p-value 9.91e-7. Module B is enriched in cellular response to calcium ion with p-value 4.04e-7. Module D is enriched in MAP kinase activity with p-value 8.60e-5. Patterns I and II are two specific interaction patterns in the subnetwork.

Figure 5 illustrates an induced subnetwork discovered by SGPG from the *Homo sapien *PPI network. Only the interactions among the proteins in Figure 5 are exhibited. As shown in Figure 5, modules A, B and C are all sparsely connected modules, which have no interactions inside the modules. Proteins in module D only have a few connections. Modules A and B are annotated to KOG K (transcription). While module D is annotated to KOG T (signal transduction mechanisms). Module C is annotated to both KOG T and K. Module C contains proteins SMAD2 and SMAD3 which play an important role in tumor generation [20]. From the module structure in Figure 5, we find that SMAD2 and SMAD3 have intimate relationships to proteins of DNA binding, cellular response and kinase activity, which is useful to help us to obtain a better understanding of their functionalities and influence on other proteins. Furthermore, the two interaction patterns preserved in the module structure are detected by SGPG, which is difficult for MCL to identify.

Table 6 lists three sparsely connected module examples detected by SGPG but missed by MCL. These three modules are annotated to KOG T and K respectively, which cannot be detected by MCL no matter what inflation parameter we choose.

**Table 6:** Sparse modules in T and K KOG categories for *Homo sapien *PPI network

KOG ID	Sparse module example	Enriched genes	Enriched GO Term	GO Level	*p*-value
T	NTRK1, NTRK3, NTRK2 VAV1, VAV3	NTRK1, NTRK2, NTRK3	neurotrophin receptor activity	[+3, -1]	2.95e-9
T	PIK3R3, PIK3R2, PIK3R1	PIK3R3, PIK3R2, PIK3R1	phosphatidylinositol 3-kinase complex	[+5, -1]	4.77e-9
K	JUN, JUNB, JUND SPIB	JUN, JUNB, JUND	cellular response to calcium ion	[+6, -1]	4.04e-7

## Discussion

At present, most of the module identification methods for biological networks aim to find densely connected modules but ignore sparely connected modules, which can be manifested in biological systems due to their special functionalities. Here, in order to find more biologically meaningful modules with both types of modular structures, we adopt a blockmodel framework which detects densely connected modules and sparely connected modules simultaneously as it identifies modules by the interaction patterns. Our results indicate that the real world PPI networks, such as *Saccharomyces cerevisia *and *Homo sapien *PPI networks, do have the sparely connected modules, which may not be detected by the modularity based methods such as MCL.

We have proposed a novel efficient method SGPG that combines SG and PG to solve the blockmodel functional module identification problem. Our experimental results have proven that our SGPG method can achieve competitive performance numerically and biologically but with significantly reduced computation time compared to the original SA method in [2]. We have demonstrated that SGPG can identify biologically meaningful modules, specifically the ones with sparse interactions within them but with same interaction patterns to the rest of the network, which behave important cellular functionalities. Our future research will focus on designing more efficient algorithms to detect functional modules in large-scale biological networks. Our method can be further improved with the potential to enhance the performance. For example, the number of modules *q *needs to be given in our current algorithm. In [21], the authors have introduced a Bayesian strategy based on a stochastic block model to identify the module assignments as well as the optimal number of modules. However, this Bayesian approach only guarantees that the final solution converges to the local optimum. We may be able to combine the strengths from our SGPG method and the Bayesian approach to efficiently determine the optimal *q *in SGPG by adopting this Bayesian strategy to further improve the proposed algorithm. Also, there are some other promising efficient heuristics for global optimization, such as differential evolution [22] and genetic algorithms [23], which may also be coupled with our PG strategy to further increase the efficiency of these algorithms.

## Competing interests

The authors declare that they have no competing interests.

## Authors' contributions

Conceived the algorithm: YW, XQ. Implemented the algorithm and performed the experiments: YW. Analyzed the results: YW, XQ. Wrote the paper: YW, XQ.

## Declarations

The publication costs for this article were funded by the corresponding author's institution.

This article has been published as part of *BMC Bioinformatics *Volume 14 Supplement 2, 2013: Selected articles from the Eleventh Asia Pacific Bioinformatics Conference (APBC 2013): Bioinformatics. The full contents of the supplement are available online at http://www.biomedcentral.com/bmcbioinformatics/supplements/14/S2.
